# Effects of Repetitive Transcranial Magnetic Stimulation on Tumor Necrosis Factor Alpha in Neuropsychological Disorders: A Systematic Review and Meta‐Analysis

**DOI:** 10.1002/brb3.70329

**Published:** 2025-02-11

**Authors:** Arya Asadizeidabadi, Seyedmohammadamin Hosseini, Artem Pyatkov

**Affiliations:** ^1^ Universal Scientific Education and Research Network (USERN) Tehran Iran; ^2^ Sechenov University Moscow Russia; ^3^ Department of Functional Diagnostics The Clinic of Nervous Diseases Named After. A.Ya. Kozhevnikov University Clinical Hospital No. 3 Moscow Russia

**Keywords:** brain‐derived neurotrophic factor (BDNF) | inflammatory cytokine | repetitive transcranial magnetic stimulator | tumor necrosis factor‐alpha (TNF‐α)

## Abstract

**Introduction:**

Repetitive transcranial magnetic stimulation (rTMS) shows promise in treating neuropsychological disorders. This systematic review and meta‐analysis investigated the effects of rTMS on inflammatory cytokines and brain‐derived neurotrophic factor (BDNF) under these conditions.

**Method:**

We searched five electronic databases for relevant studies. Twelve studies (six randomized controlled trials [RCTs] and six cohort studies) were included in the systematic review, with six RCTs included in the meta‐analysis. The primary outcome was tumor necrosis factor‐alpha (TNF‐α) levels, with secondary outcomes including interleukin‐6 (IL‐6), interleukin‐1 beta (IL‐1β), high‐sensitivity C‐reactive protein (hs‐CRP), interferon‐gamma (IFN‐γ), and BDNF.

**Results:**

Meta‐analysis revealed a significant decrease in TNF‐α levels after rTMS (weighted mean difference [WMD]: −6.65, 95% confidence interval [CI]: −10.47–2.83, *p *< 0.05), with greater effects associated with longer interventions. No significant change was found in the IL‐6 levels. BDNF levels increased significantly (WMD: 7.97, 95% CI: 2.8–13.15, *p* < 0.05). Qualitative synthesis indicated consistent reductions in IL‐1β. High heterogeneity was observed in some analyses.

**Conclusion:**

rTMS may exert therapeutic effects on neuropsychological disorders partly through modulating neuroinflammation and promoting neuroplasticity. However, high heterogeneity and study limitations necessitate larger, more standardized clinical trials to confirm these effects and explore their clinical significance.

## Graphical abstract

Repetitive transcranial magnetic stimulation (rTMS) shows a significant negative correlation with TNF‐a levels, suggesting its potential to reduce neuroinflammation. No significant correlation was observed with IL‐6 levels, while a positive association was found with BONF.

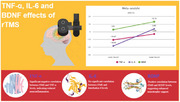

## 1 Introduction

Repetitive transcranial magnetic stimulation (rTMS) is an FDA‐approved therapy that has been effective for patients with major depressive disorder (MDD) since 2008 (O'Reardon et al. 2007). rTMS is a noninvasive therapeutic tool that employs rapidly alternating electric current through a circular coil positioned close to the scalp head to create a magnetic field that in turn produces electrical field in the cortex to influence neuron activity and promote neuroplasticity (Richter, Kellner, and Licht 2023). rTMS is a treatment option for neurological and neuropsychological disorders, such as MDD (Miron et al. 2021), poststroke cognitive impairment (PSCI) (Gong et al. 2023), and neuropathic pain (Tsai et al. 2023) and for pain management (Yang and Chang 2020). rTMS has the ability to regulate a variety of brain pathways, including those involving growth factors, consistent hormones, and inflammation (Padberg et al. 2002). Cytokines are small proteins that are released by cells throughout the body but are released mainly by immune cells. They are directly involved in signal initiation for the inflammatory response and control the inflammatory process (Zhang and An 2007). Tumor necrosis factor alpha (TNF‐α) is a cytokine or signaling protein recognized to be involved in the control of immune cells and inflammation. It participates in many homeostatic and inflammatory functions, making it a standout player in autoimmune and inflammatory diseases (Gonzalez Caldito 2023). TNF‐α plays a central role as a mediator of acute and chronic inflammation. Under certain conditions, it can activate cell death via apoptosis and necroptosis (van Loo and Bertrand 2023). In addition to causing systemic inflammation, TNF‐α is normally and abnormally involved in many physiological and pathological processes in the CNS (Gonzalez Caldito 2023). TNF‐α is implicated in several situations, including neuroinflammation (Subedi et al. 2020), mood disorders (Uzzan and Azab 2021), cognitive dysfunction (Hennessy et al. 2017), neuropathic pain (Leung and Cahill 2010), and disruption of the blood‒brain barrier (Gonzalez Caldito 2023). To date, few studies have investigated the overall effect of rTMS on inflammatory cytokines in the form of systematic reviews and meta‐analyses. Consequently, our study addresses this lack of awareness by targeting the shift in inflammatory cytokines, with particular attention to TNF‐α, in neurological and neuropsychological patients receiving rTMS treatment. The focus of our study was to provide useful information about the impact of rTMS on inflammation and related pathways, which will increase the understanding of its benefits and risks in different diseases.

## 2 Methods

The Preferred Reporting Items for Systematic Reviews and Meta‐Analyses (PRISMA) guidelines (Page et al. 2021), the Cochrane Handbook for Systematic Reviews of Interventions (Nasser 2020), and the protocol was registered in PROSPERO (Schiavo 2019) with the following link: PROSPERO record CRD42024524980.

### 2.1 Data Sources and Search Strategy

From the beginning to February 17, 2024, a thorough search was performed via five English electronic databases: PubMed, the Cochrane Library, Web of Science, ClinicalTrials.gov, and Scopus. MeSH terms and keywords related to “tumor necrosis factor” and “transcranial magnetic stimulation” were included in the search strategy. Supporting Information Appendix A and Table S1 contain specific directions for collecting data from PubMed and other databases.

### 2.2 Inclusion and Exclusion Criteria

Studies that satisfied the following “PICOS” criteria (Moher, Liberati, Tetzlaff, Altman, and Group 2009) were found to be suitable for inclusion: (1) P (population): participants who were diagnosed with various types of neuropsychological diseases (e.g., depression and postischemic stroke depression) and neurological diseases (e.g., poststroke aphasia and neuropathic pain). (2) I (intervention): TMS. (3) C (control): The types of controls involve treatment as usual control, Sham‐rTMS, or another medication indicated for neurological conditions (e.g., myofascial pain syndrome) and neuropsychological patients. This means that patients in the control group will not receive treatments similar to those in the intervention groups until the end of the trial. (4) O (outcome): The main outcome was any significant effect on the quantity of TNF‐α. The additional outcomes were any significant effects on the quantity of interferon‐gamma (IFN‐γ), interleukin‐6 (IL‐6), interleukin‐1 beta (IL‐1β), high‐sensitivity C‐reactive protein (hc‐CRP), or brain‐derived neurotrophic factor (BDNF). (5) S (study design): For systematic review and meta‐analysis, randomized controlled trials (RCTs) were included, whereas cohorts were utilized exclusively for systematic review.

### 2.3 Screening Procedure

We used EndNote 21 (Clarivate Analytics, USA) to manage the primary search results to find and remove duplicates. Two independent reviewers (A.A. and S.H.) identified articles meeting the inclusion criteria by using abstracts and titles and checked the full texts of the studies. Solutions to these disputes were provided by a third checker (A.P.) during these procedures.

### 2.4 Risk‐of‐Bias Evaluation

The quality of the included studies was assessed via the Joanna Briggs Institute (JBI) tool (Moola et al. 2015). The JBI scale comprises 13 items for RCT, 11 each with responses of YES (positive rating), NO (negative rating), N/A (not applicable), or unclear. Studies are categorized as high risk if they score below a specified threshold, typically approximately 50% or lower; moderate risk if they fall between 50% and 75%; and low risk if their scores exceed 75%. The quality assessment was carried out by two authors (A.A. and S.H.) separately, and a final consensus was reached afterward.

### 2.5 Data Extraction

Data extraction was performed via forms created alongside this protocol on a Microsoft Excel platform. The specific data collected from every trial included author‐associated information, specific title of the study, country of origin, year of publication, type of study carried out, total number of individuals in the sample, demographics of the participants, details of the intervention, frequency and duration, place where the intervention was made, outcomes, follow‐up sessions, adverse effects, and risk of bias.

### 2.6 Data Synthesis and Analysis

Meta‐analyses were conducted via the STATA 14 software (StataCorp. LLC) program tool. In the network of studies, whether regarding continuous or categorical variables, the typical analysis metric used for meta‐analysis was the mean change and standard error (SE) difference. The standard difference (SD) at baseline and the last intervention in SE and the mean value at baseline and the last mean at the last intervention in the case of MD were employed in the analysis. Additionally, we converted SD to SE via the following formula:

SE=SD/√n

The program calculates the weighted mean difference (WMD) and confidence interval (CI) via WMD and SE. Significant effects were determined by a *p* value <0.05 or CI excluding zero. The statistics and *p* value of heterogeneity were used to assess the level of heterogeneity in the studies. Heterogeneity in the $I^{2}$ was classified as follows: 0%–40%, not important; 30%–60%, moderate; and 50%–90%, and substantial, with 75%–100%, indicating considerable heterogeneity (Higgins et al. 2023). Additionally, heterogeneity in the *p* value was classified as follows: >0.05, indicating no heterogeneity. We utilized regression analysis to identify the source of heterogeneity, followed by subgroup analyses to reduce the statistical significance and increase the *p* value. We utilized a sensitivity test to assess the impact of individual studies on the overall results.

### 2.7 Assessment of Publication Bias

Publication bias was checked via a funnel plot along with Egger's test. For Egger's test, we calculated the *p* value of the SE of effect size (ES), and we created a funnel plot to check for publication bias (see Supporting Information Appendix B, Table S2).

## 3 Results

### 3.1 Search Results

**FIGURE 1:**
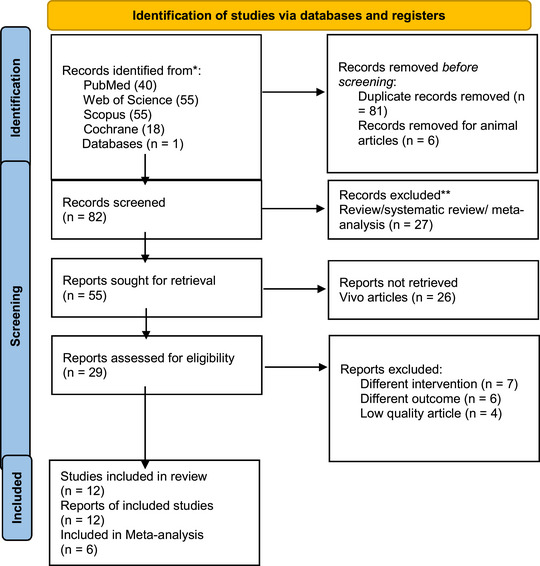
PRISMA flowchart diagram of the study (Page et al. 2021). *If feasible, consider reporting the number of records identified from each database or register searched (rather than the total number across all databases/registers). ** If automation tools were used, indicate how many records were excluded by a human and how many were excluded by automation tools. PRISMA, Preferred Reporting Items for Systematic Reviews and Meta‐Analyses.

Initially, 169 results were obtained from five databases. Two reviewers (A.A. and S.H.) independently subsequently conducted the screening procedure. In the first step, 81 duplicate articles were excluded. Next, they deleted 32 in vivo articles and 27 systematic reviews, reviews, and meta‐analyses on the basis of the title and abstract of the results. Following this initial screening, the screening tools carefully reviewed the full texts of 55 articles. Six RCTs (Bai et al. 2022; Liu et al. 2022; Medeiros et al. 2016; Wang et al. 2022; Zhang, Xue et al. 2020; Zhao Li, Tian, Zhu, and Zhao 2019) and six cohort studies (Boylu et al. 2023; Cha et al. 2022; Tateishi et al. 2020; Valiuliene et al. 2021; Wu and Liu 2023; Yilmaz et al. 2022) were considered suitable for inclusion in this systematic review, and six studies were included in the meta‐analysis. Figure 1 illustrates the literature retrieval and screening procedure.

### 3.2 Risk of Bias

To assess the quality of the studies included in our research, we utilized the JBI tools (Moola et al. 2015). To review the studies, we used checklists: one for RCTs and one for cohort studies. These evaluations resulted in all of the studies for which the intervention was categorized as having low risk, whereas for one study, moderate risk was considered (Bai et al. 2022). Additionally, the highest RCT score was for one study (Wang et al. 2022), which scored the highest RCT score, which received 12 out of 13 “Yes” answers, whereas the other study (Bai et al. 2022) had the lowest score, with 8 “Yes” answers out of the 13 questions. Each study was scored against the 11 questions related to cohort studies. Among the 11 questions for cohort studies, the maximum rating score of “Yes” given was 11 in Study (Wu and Liu 2023), whereas the minimum rating score of “Yes” was 9 in both studies (Valiuliene et al. 2021; Yilmaz et al. 2022). Table 1 shows the quality of the studies that we used for this study.

**TABLE 1:** Quality of studies.

Study	1	2	3	4	5	6	7	8	9	10	11	12	13	Overall
Medeiros et al. (2016)	**Y**	**Y**	**Y**	**Y**	**Y**	**N**	**N**	**N**	**Y**	**Y**	**Y**	**Y**	**Y**	**L**
Zhao Li, Tian, Zhu, and Zhao (2019)	**Y**	**N/A**	**Y**	**N/A**	**N/A**	**N/A**	**Y**	**Y**	**Y**	**Y**	**Y**	**Y**	**Y**	**L**
Zhang et al. (2020)	**Y**	**N/A**	**Y**	**N/A**	**N/A**	**N/A**	**Y**	**Y**	**Y**	**Y**	**Y**	**Y**	**Y**	**L**
Liu et al. (2022)	**Y**	**N/A**	**Y**	**N/A**	**N/A**	**N/A**	**Y**	**Y**	**Y**	**Y**	**Y**	**Y**	**Y**	**L**
Wang et al. (2022)	**Y**	**Y**	**Y**	**Y**	**Y**	**N**	**Y**	**Y**	**Y**	**Y**	**Y**	**Y**	**Y**	**L**
Bai et al. (2022)	**Y**	**N/A**	**Y**	**N/A**	**N/A**	**N/A**	**Y**	**N**	**Y**	**Y**	**Y**	**Y**	**Y**	**M**
Tateishi et al. (2020)	**N**	**Y**	**Y**	**Y**	**Y**	**Y**	**Y**	**Y**	**Y**	**Y**	**Y**	**—**	**—**	**L**
Valiuliene et al. (2021)	**Y**	**Y**	**Y**	**Y**	**Y**	**Y**	**Y**	**Y**	**N**	**N**	**Y**	**—**	**—**	**L**
Cha et al. (2022)	**N**	**Y**	**Y**	**Y**	**Y**	**Y**	**Y**	**Y**	**Y**	**Y**	**Y**	**—**	**—**	**L**
Yilmaz et al. (2022)	**N**	**Y**	**Y**	**Y**	**Y**	**Y**	**Y**	**Y**	**N**	**Y**	**Y**	**—**	**—**	**L**
Wu and Liu (2023)	**Y**	**Y**	**Y**	**Y**	**Y**	**Y**	**Y**	**Y**	**Y**	**Y**	**Y**	**—**	**—**	**L**
Boylu et al. (2023)	**N**	**Y**	**Y**	**Y**	**Y**	**Y**	**Y**	**Y**	**Y**	**Y**	**Y**	**—**	**—**	**L**

Abbreviations: L, low; M, moderate; N, no; N/A, not recommend; Y, yes.

### 3.3 Study Characteristics

The studies are composed of six RCTs (e.g., Bai et al. 2022; Liu et al. 2022; Medeiros et al. 2016; Wang et al. 2022; Zhang, Xue et al. 2020; Zhao et al. 2019), and all originated from China (Bai et al. 2022; Liu et al. 2022; Wang et al. 2022; Zhang, Xue et al. 2020; Zhao et al. 2019), with the exception of one from Brazil/the United States (Medeiros et al. 2016). Among these RCTs, two trials targeted depressive patients (Wang et al. 2022; Zhao et al. 2019), two trials targeted poststroke depression patients (Liu et al. 2022; Zhang, Xue et al. 2020), one trial targeted myofascial pain syndrome patients (Medeiros et al. 2016), one targeted poststroke aphasia patients (Bai et al. 2022), and one targeted poststroke aphasia patient (36). In addition, six cohort studies and one targeted poststroke aphasia patient were included (Bai et al. 2022). In addition, six cohort studies (Boylu et al. 2023; Cha et al. 2022; Tateishi et al. 2020; Valiuliene et al. 2021; Wu and Liu 2023; Yilmaz et al. 2022) conducted between 2020 (Tateishi et al. 2020) and 2023 (Boylu et al. 2023; Wu and Liu 2023) were included. Two were from Turkey (Boylu et al. 2023; Yilmaz et al. 2022), one from China (Wu and Liu 2023), one from South Korea (Cha et al. 2022), one from Lithuania (Valiuliene et al. 2021), and one from Japan (Tateishi et al. 2020). Among them, three focused on treatment‐resistant depression (Tateishi et al. 2020; Valiuliene et al. 2021; Yilmaz et al. 2022), one focused on depression (Boylu et al. 2023), one focused on PSCI (Cha et al. 2022), and one focused on neuropathic pain (Wu and Liu 2023). The mean age of the participants ranged from 42 (Yilmaz et al. 2022) to 65 (Zhao et al. 2019) years, and the duration of interventions varied from 10 days (Medeiros et al. 2016) to 2 months (Boylu et al. 2023; Liu et al. 2022). Eight of the studies utilized a 10 Hz frequency (Boylu et al. 2023; Medeiros et al. 2016; Tateishi et al. 2020; Valiuliene et al. 2021; Wang et al. 2022; Wu and Liu 2023; Zhang, Xue et al. 2020; Zhao et al. 2019), whereas two studies used a 20 Hz frequency (Cha et al. 2022; Yilmaz et al. 2022). Furthermore, one study used a 1 Hz frequency (Bai et al. 2022), and the other used a 0.5 Hz frequency (Liu et al. 2022). Additionally, in eight of them, the intervention targeted the left dorsolateral prefrontal cortex (DLPFC) (Boylu et al. 2023; Cha et al. 2022; Liu et al. 2022; Tateishi et al. 2020; Valiuliene et al. 2021; Wang et al. 2022; Yilmaz et al. 2022; Zhao et al. 2019), whereas in other studies, it targeted the right hemisphere (RH) (Bai et al. 2022), top of the head (Zhang, Xue et al. 2020), left motor cortex (LMC) (Medeiros et al. 2016), and primary motor cortex (M1) (Wu and Liu 2023). All studies measured TNF‐α (Bai et al. 2022; Boylu et al. 2023; Cha et al. 2022; Liu et al. 2022; Medeiros et al. 2016; Tateishi et al. 2020; Valiuliene et al. 2021; Wang et al. 2022; Wu and Liu 2023; Yilmaz et al. 2022; Zhang, Xue et al. 2020; Zhao et al. 2019), eight studies measured IL‐6 (Cha et al. 2022; Liu et al. 2022; Medeiros et al. 2016; Tateishi et al. 2020; Wang et al. 2022; Wu and Liu 2023; Yilmaz et al. 2022; Zhang, Xue et al. 2020), six studies measured IL‐1β (Boylu et al. 2023; Cha et al. 2022; Liu et al. 2022; Tateishi et al. 2020; Wu and Liu 2023; Yilmaz et al. 2022; Zhao et al. 2019), and four studies measured hs‐CRP (Cha et al. 2022; Wang et al. 2022; Yilmaz et al. 2022; Zhang, Xue et al. 2020). Additionally, two studies measured IFN‐γ (Wang et al. 2022; Yilmaz et al. 2022), and six studies measured BDNF (Bai et al. 2022; Boylu et al. 2023; Medeiros et al. 2016; Valiuliene et al. 2021; Yilmaz et al. 2022; Zhao et al. 2019). All the studies assessed these inflammatory cytokines in the serum and blood. The features of the studies that are included in this article are listed in Table 2 (for more detailed information, including the Supporting Information section in Excel format).

**TABLE 2:** Characteristics of included studies.

Study/Country	Study design	Patients	Sample size	Sex (M/F)	Age (mean)	Intervention duration (days)	Medication usage	rTMS pulse frequency (Hz)	Place of intervention	Inflammatory Cytokines Measured
Medeiros et al. (2016) Brazil and the United States	RCT	Myofascial pain syndrome	T: 46 I: 12 P: 11	T: 0/46	I: 45.83 P: 46.73	10	I: Analgesic drug, rTMS CNS medication (8)	10	LMC	TNF‐α, IL‐6
Zhao et al. (2019) China	RCT	Depression	T: 58 I: 29 P: 29	I: 16/13 C: 15/14	I: 65.97 C: 65.48	30	T: Venlafaxine Alprazolam	10	DLPFC	IL‐1β, TNF‐α
Zhang et al. (2020) China	RCT	Poststroke depression	T: 113 I: 57 C: 56	I: 36/21 C: 33/23	I: 63.67 C: 63.28	30	N/A	10	Top of head	IL‐6, TNF‐α, hs‐CRP
Liu et al. (2022) China	RCT	Poststroke depression	T: 70 I: 35 C: 35	I: 23/12 C: 20/15	I: 50.20 C: 55.61	60	T: OI, anticoagulant, antiplatelet C: antidepressant paxil hydrochloride	0.5	DLPFC	IL‐1β, IL‐6, TNF‐α
Wang et al. (2022) China	RCT	Depression	T: 57 I: 29 P: 28	I: 5/24 P: 6/22	I: 58.2 P: 55.6	14	T: non‐benzodiazepine antidepressants	10	DLPFC	TNF‐α, IFN‐γ, IL‐6, hs‐CRP
Bai et al. (2022) China	RCT	Poststroke aphasia	T: 60 I: 30 P: 30	I: 17/13 P: 14/16	I: 63.47 P: 59.91	30	N/A	1	RH	TNF‐α
Tateishi et al. (2020) Japan	Cohort	Treatment‐resistant depression	I: 11	I: 6/5	I: 52.2	45	I: Antidepressant	10	DLPFC	IL‐1β, IL‐6, TNF‐α
Valiuliene et al. (2021) Lithuania	Cohort	Treatment‐resistant depressive	I: 20 HC: 18	Primary sample size before dropout: I:10/15 HC: 4/15	I: 48.04 HC: 45.53	45	I: non‐benzodiazepine antidepressants	10	DPFC	TNF‐α
Cha et al. (2022) South Korea	Cohort	Poststroke cognitive impairment	I: 10 HC: 11	I: 8/2 HC: 8/3	I: 53.8 HC: 61.8	14	N/A	20	DLPFC	TNF‐α, IL‐1β, IL‐6, hs‐CRP
Yilmaz et al. (2022) Turkey	Cohort	Treatment‐resistant depression	I: 10 HC: 9	I: 4/6 HC: 4/5	I: 42 HC: 45	7	I: Atypical antipsychotic medication	20	DLPFC	TNF‐α, IL‐6, IFN‐γ, hs‐CRP
Wu and Liu (2023) China	Cohort	Neuropathic pain	I: 31 C: 30	I: 15/16 C: 16/14	I: 57.45 C: 57.23	30	N/A	10	M1	TNF‐α, IL‐1β, IL‐6
Boylu et al. (2023) Turkey	Cohort	Depressive	I: 23 C: 25	I: 8/15 C: 10/15	I: 32.09 C: 31.44	60	N/A	10	DLPFC	TNF‐α, IL‐1β

Abbreviations: C, control; CNS, central nervous system; DLPFC, left dorsolateral prefrontal cortex; F, female; HC, healthy control; hs‐CRP, high‐sensitivity C‐reactive protein; Hz, hertz; I, intervention; IFN‐γ, interferon‐gamma; IL‐1β, interleukin‐1 beta; IL‐6, interleukin‐6; LMC, left motor cortex; M, male; M1, primary motor cortex; OI, oxygen inhalation; P, placebo; RCT, randomized controlled trial; RH, right hemisphere; rTMS, repetitive transcranial magnetic stimulation; T, total; TNF‐α, tumor necrosis factor‐alpha.

### 3.4 Effects on Inflammatory Cytokines

All studies included in this study measured TNF‐α. Among them, eight studies reported a significant decrease in TNF‐α levels (Bai et al. 2022; Boylu et al. 2023; Cha et al. 2022; Liu et al. 2022; Wang et al. 2022; Wu and Liu 2023; Yilmaz et al. 2022; Zhang, Xue et al. 2020; Zhao et al. 2019), three studies reported no significant decrease (Medeiros et al. 2016; Tateishi et al. 2020; Valiuliene et al. 2021), and one study did not report these results (Bai et al. 2022). Among the 3 studies that reported no significant decrease, 2 had a small sample size (10 participants in the intervention group) (Tateishi et al. 2020; Valiuliene et al. 2021), and 1 focused specifically on a female population (Medeiros et al. 2016). Eight studies measured IL‐6 levels, with four reporting significant changes and four reporting no significant changes. Two of the studies that reported no significant changes had small sample sizes (Tateishi et al. 2020; Yilmaz et al. 2022), whereas the other two studies with no significant changes had a high proportion of female patients in their sample sizes (Medeiros et al. 2016; Wang et al. 2022). Six studies measured IL‐1b, and all of them reported a significant change in this inflammatory cytokine. Four studies reported on hs‐CRP levels, with half of them showing a significant decrease and the other half showing no significant changes. The two studies that reported no changes had small sample sizes (Cha et al. 2022; Yilmaz et al. 2022). Two studies reported IFN‐γ levels. One study revealed a significant decrease, whereas the other did not. The study that showed no changes had a small sample size (Yilmaz et al. 2022).

### 3.5 Effect on BDNF

Six studies reported on BDNF, four of which reported a significant increase, whereas two studies reported no significant change. The study that demonstrated no significant change had a small sample size (Yilmaz et al. 2022), and the other study, which also showed no significant change, included only a female population (Medeiros et al. 2016).

### 3.6 Data Synthesis

Six RCTs were included in this meta‐analysis, all of which were conducted in China, except for one that was conducted in the United States/Brazil. Among these studies, two focused on depression, two focused on poststroke depression, one focused on patients with myofascial pain syndrome, and the other focused on poststroke aphasia patients. Additionally, the sites of intervention varied: Three studies targeted the DLPFC, one focused on the LMC, and one each targeted the RH. Notably, all the studies utilized 10 Hz frequency pulses, except for two studies, which used 1 and 0.5 Hz pulses. Moreover, the intervention duration ranged from 2 months to 10 days. Three comparator groups received placebos, whereas the remaining three had control groups. The sample was composed of an intervention group of 190 patients and an equivalent control group of 183 participants. The current comprehensive theoretical review was undertaken with the help of a more advanced random‐effects model and quantitatively evaluated via the JBI tool. Table 3 presents the specific attributes of each of the included studies.

**TABLE 3:** Features of the studies in the meta‐analysis that was included.

						Sex (%)		Sample size		Analysis type		
Study/Country	Patient	Intervention place	rTMS pulse frequency (Hz)	Control group	Duration (days)	Women	Men	IG	CG	M1	M2	M3
Medeiros et al. (2016)/Brazil/the United States	Myofascial pain syndrome	LMC	10	Placebo	10	100	0	12	11	Y	Y	Y
Zhao et al. (2019)/China	Depression	DLPFC	10	Control	30	47	53	29	29	Y	N	Y
Zhang et al. (2020)/China	Poststroke depression	Top of head	10	Control	30	29	61	57	56	Y	Y	N
Liu et al. (2022)/China	Poststroke depression	DLPFC	0.5	Control	60	29	61	35	35	Y	Y	N
Wang et al. (2022)/China	Depression	DLPFC	10	Placebo	14	80	20	29	28	Y	Y	N
Bai et al. (2022)/China	Poststroke aphasia	RH	1	Placebo	30	49	51	28	24	N	N	Y

Abbreviations: CG, control group; DLPFC, left dorsolateral prefrontal cortex; IG, intervention group; LMC, left motor cortex; M1, Measure 1 (tumor necrosis factor‐alpha); M2, Measure 2 (interleukin‐6); M3, Measure 3 (Brain‐derived neurotrophic factor); N, no.; RH, right hemisphere; Y, yes.

### 3.7 Tumor Necrosis Factor‐Alpha

Five studies were included in the TNF‐α meta‐analysis. We used the WMD for this analysis and conducted a subgroup meta‐analysis on the basis of intervention time. Two studies had intervention times of less than 1 month (10 and 14 days), whereas three studies had intervention times of more than 1 month (two studies for 1 month and one study for 2 months).

**FIGURE 2:**
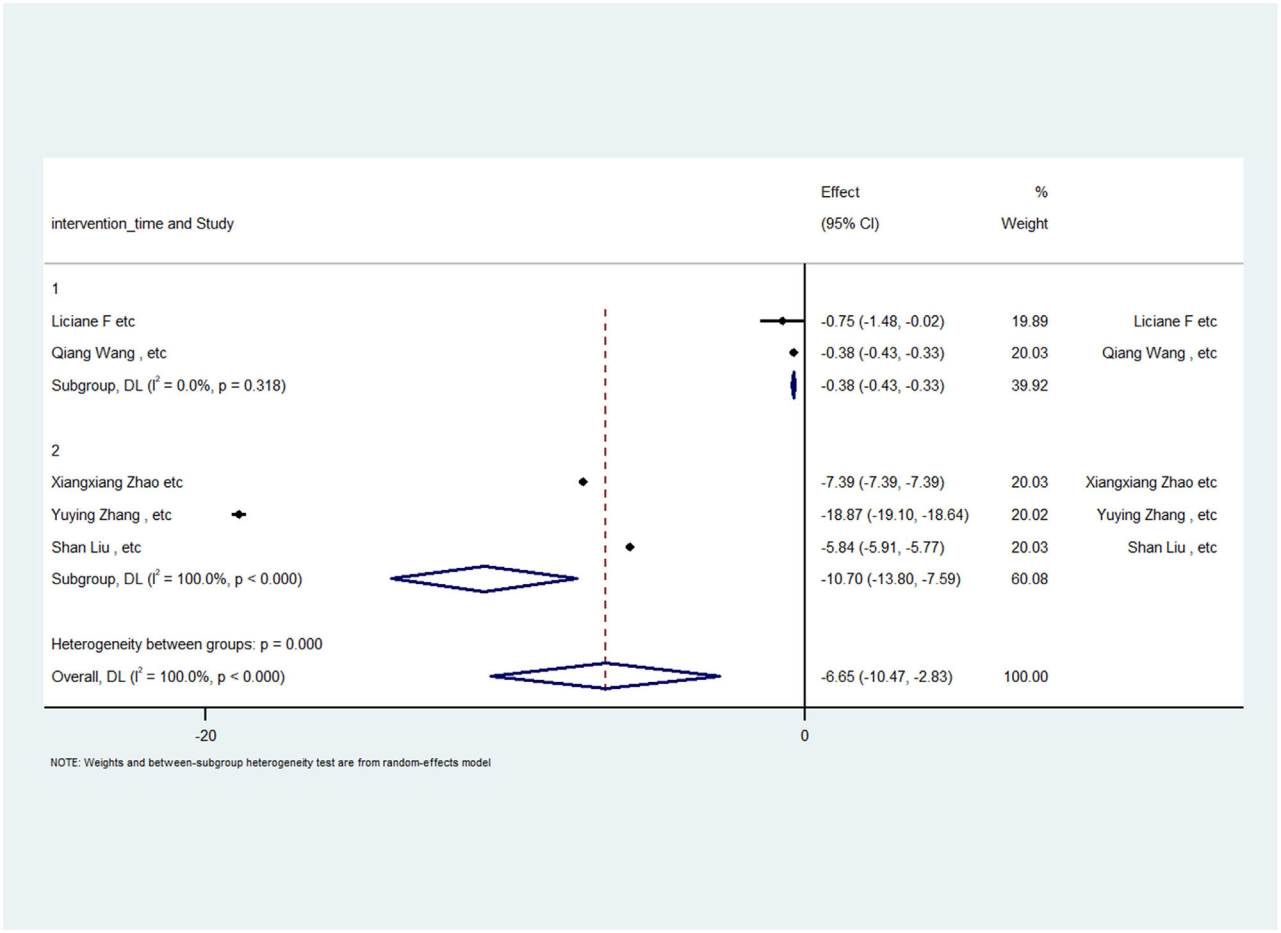
Forest plot and meta‐analysis of TNF‐alpha categorized by intervention time.

**FIGURE 3:**
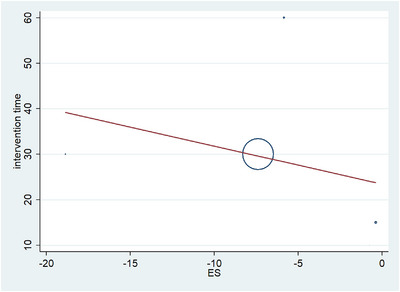
Meta‐regression between intervention time and effect size of TNF‐α.

The analysis revealed a WMD of −0.38 with a CI of −0.42 to −0.33 for studies with intervention times under 1 month, and for studies with intervention times over 1 month, the WMD was −10.7 with a CI of −13.8 to −7.59. The overall WMD was −6.65, with a CI of −10.47 to −2.83. Studies with intervention times less than or equal to 1 month demonstrated significant changes in the *p* value (*p* value < 0.05), as did studies with intervention times longer than or equal to 1 month (*p* value < 0.05). Additionally, the overall *p* value significantly changed (*p* value < 0.05). Furthermore, heterogeneity in the statistics revealed no heterogeneity in the group with more than 1 month of intervention ($I^{2}$ = 0.0%), whereas for the group with more than 1 month of intervention, there was high heterogeneity ($I^{2}$ = 100%). The overall heterogeneity statistic also indicated high heterogeneity in the results ($I^{2}$ = 100%). Table 4 shows the results, and a forest plot is displayed in Figure 2. In the forest plot, the data on separate studies and the total meta‐analysis effect are shown. The horizontal lines in each graph represent a single study, where the square represents the ES and the line going up or down represents 95% CI. The size of each square is proportional to the weight of the study in the meta‐synthesis. The diamond at the bottom reflects the overall measure of ES across all the findings. Values to the right of the vertical zero line mean an increase in TNF‐α levels, whereas the values to the left of the line imply decreased levels. If the diamond crosses both sides of the zero line, it suggests no statistically significant correlation. A regression plot that gives us a statistical sense of the relationship between two variables (ES vs. intervention time on the *x* and *y* axes, respectively) is shown in Figure 3. The red diagonal line running through the plot is called a regression line, which shows the overall trend or pattern in the data: With increasing ES values, one can see that decreasing intervention time. In order to easily understand the data portrayed by this figure, it is necessary to mention that the plot presents four individual data points, not the distinctly circled one in the center. Although one of the data points is heightened with a large circle around approximately ES = −7, there are three other data points that may less instantly apparent: one at ES = −20, another at ES = 0, and a third at ES = −2.

**TABLE 4:** The effect of intervention duration on tumor necrosis factor‐alpha (TNF‐α).

				Heterogeneity	
	Number of studies	WMD (95% CI)	*p* value	P heterogeneity	*I* ^2^
Overall effect	5	−6.652 (−10.473 to −2.831)	0.000	0.000	100
30 d < ID	2	−0.378 (−0.427 to −0.328)	0.000	0.318	0.0
30 d ≥ ID	3	−10.696 (−13.799 to −7.592)	0.001	0.000	100

Abbreviations: CI, confidence interval; d, days; ID, intervention duration; WMD, weighted mean differences.

### 3.8 Interleukin‐6

**FIGURE 4:**
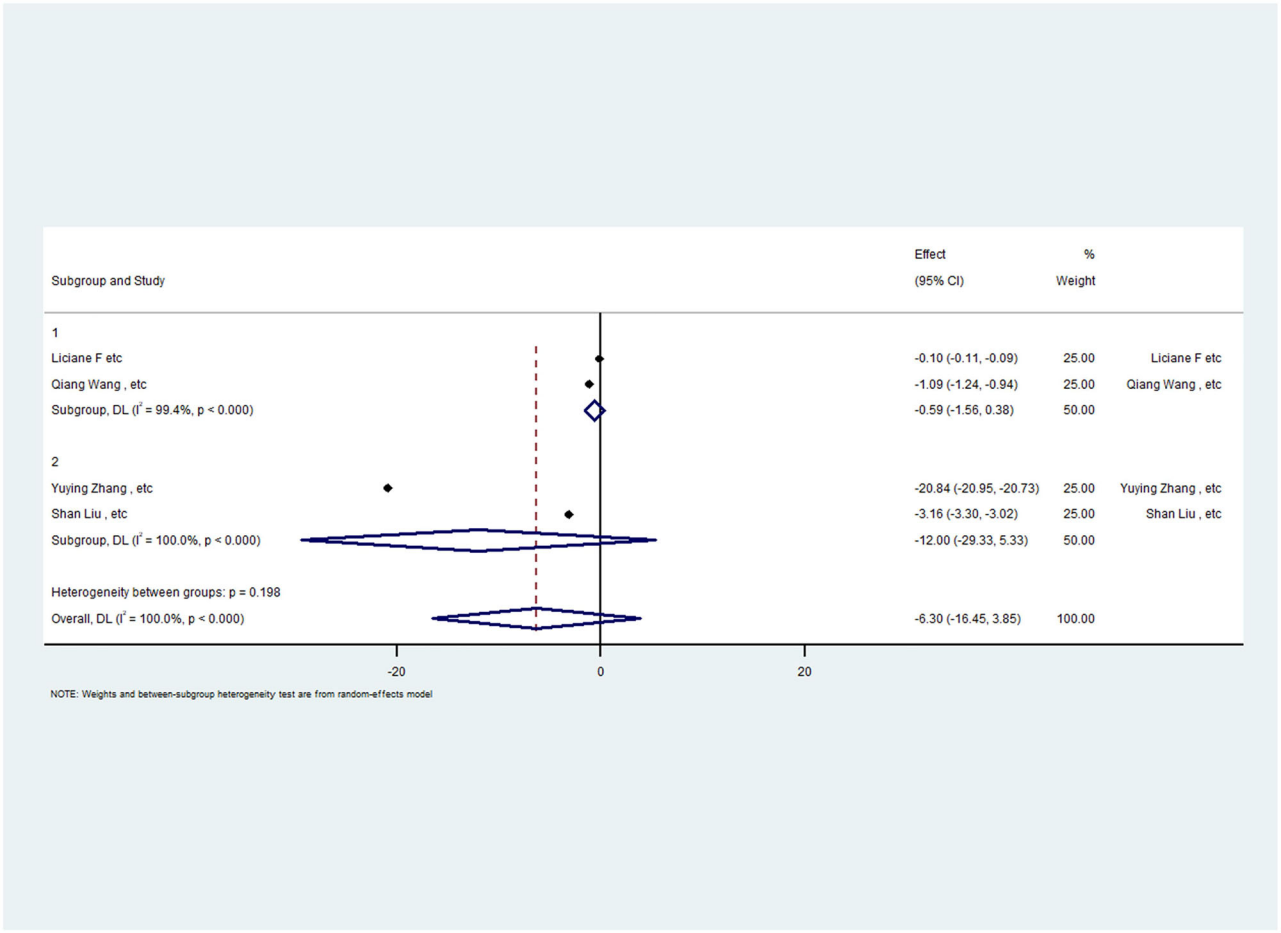
Forest plot and meta‐analysis of IL‐6 categorized by sex difference.

Four studies with WMDs and CIs were performed in the meta‐analysis, divided into two subgroups based on gender distribution. Group 1, which had a higher proportion of female participants, showed no significant correlation (*p* value > 0.05) between rTMS and IL‐6 levels (CI: −1.56, 0.38). Similarly, Group 2, with a male‐dominant population, also demonstrated no significant correlation (*p* value > 0.05, CI: −29.33, 5.33). Heterogeneity was high in both subgroups (*I* ^2^ = 100%). The overall analysis, however, showed no significant change (*p* > 0.05) for a CI of −16.45–3.85 and showed high heterogeneity (*I* ^2^ = 100%). Table 3 reports the detailed gender distribution for each study, and Table 5 contains the comprehensive statistical results. Additionally, a forest plot of this meta‐analysis is shown in Figure 4. Similarly, the IL‐6 forest plot follows the same structure as previous forest plots.

**TABLE 5:** The effect of repetitive transcranial magnetic stimulation (rTMS) on IL‐6 and BDNF.

					Heterogeneity	
		Number of studies	WMD (95% CI)	*p* value	P heterogeneity	*I* ^2^ (%)
IL‐6	Overall effect	4	−6.298 (−16.448, 3.853)	0.224	0.000	100
	Women > Men	2	−0.59 (−1.56, 0.38)	0.232	0.000	99.4
	Men > Women	2	−12.00 (−29.33, 5.33)	0.175	0.000	100
BDNF	Overall effect	3	7.974 (2.803, 13.146)	0.003	0.000	93.2

Abbreviations: BDNF, brain‐derived neurotrophic factor; CI, confidence interval; d, days; ID, intervention duration; IL‐6, interleukin‐6; WMD, weighted mean differences.

### 3.9 Brain‐Derived Neurotrophic Factor

**FIGURE 5:**
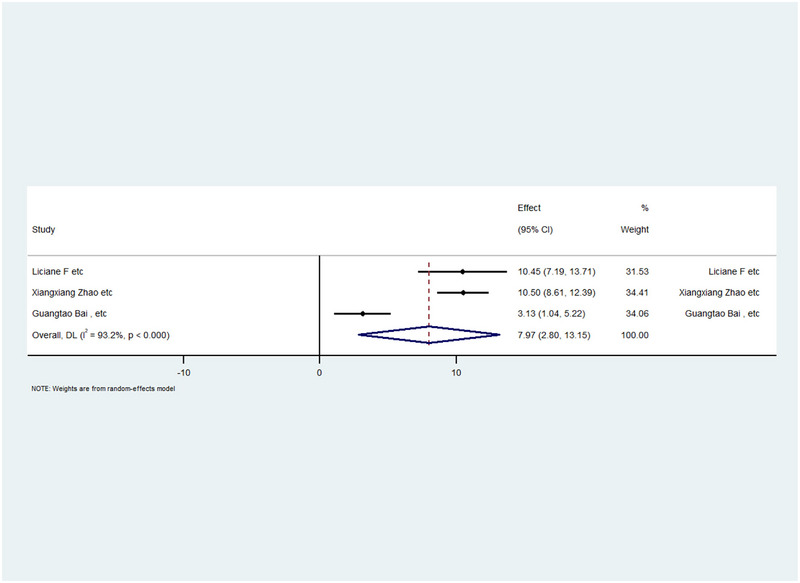
Forest plot and meta‐analysis of BDNF.

For the BDNF meta‐analysis, three studies were included. We utilized the WMD with CIs to analyze the data. The WMD was 7.97, with a CI of 2.8–13.15. However, the resulting *p* value indicated significant changes (*p* value < 0.05). Furthermore, high heterogeneity was observed in the $I^{2}$ statistic ($I^{2}$  = 93.2%). The results can be seen in Table 5. Additionally, a forest plot of this meta‐analysis is shown in Figure 5. Moreover, the BDNF forest plot shows the same content as previous forest plots.

### 3.10 Publication Bias

To examine publication bias, we used ES and SE to perform Egger's test. A *p* value less than 0.05 indicates that there is no significant source of reporting bias across the included studies. First and foremost, there is a very low risk of publication bias for each of the meta‐analyses conducted in our study. Furthermore, it was used to determine the extent to which each study adjusted the overall results of the analysis, together with sensitivity analysis for each analysis. This meant having to estimate CIs ranging between positive and negative numbers. We found that only Medeiros et al. (2016) had a statistically significant effect on the total TNF‐α concentration. Additional information is provided in Supporting Information Appendix C (Figures S1–S3, Tables S3 and S4).

### 3.11 Adverse Events (AEs)

Two of the 15 studies reviewed in this article reported AEs in patients receiving TMS therapy (Cha et al. 2022; Zhang, Xue et al. 2020). Depression patients who receive postischemic stroke treatment suffer from AEs such as constipation and hyperglycemia. However, among patients with PSCI, only headaches and insomnia have been reported. More such interventions should be employed cautiously in health care facilities; every step should be taken to counter any undesired effects.

## 4 Discussion

### 4.1 Summary of Main Findings

The purpose of the present systematic review and meta‐analysis was to synthesize findings about the effects of rTMS on TNF‐α, other inflammatory cytokines, and BDNF. To the best of our knowledge, this is the first systematic review of the effects of rTMS on TNF‐α and other cytokines and the first meta‐analysis of the effects of rTMS on TNF‐α, IL‐6, and BDNF in neuropsychological patients. It is therefore crucial to understand the impact of rTMS to design appropriate interventions to improve the quality of life of patients. As the findings of this study revealed the effects of rTMS on TNF‐α, this research also filled the literature gap. Additionally, this research examined how the effectiveness of rTMS treatment affected IL‐6, IL‐1β, hs‐CRP, IFN‐γ, and BDNF in participants, and our study revealed that rTMS was significantly correlated with various inflammatory cytokines. Importantly, our following studies did not reveal any severe side effects of rTMS therapy.

### 4.2 TNF‐α and Other Inflammatory Cytokines

**FIGURE 6:**
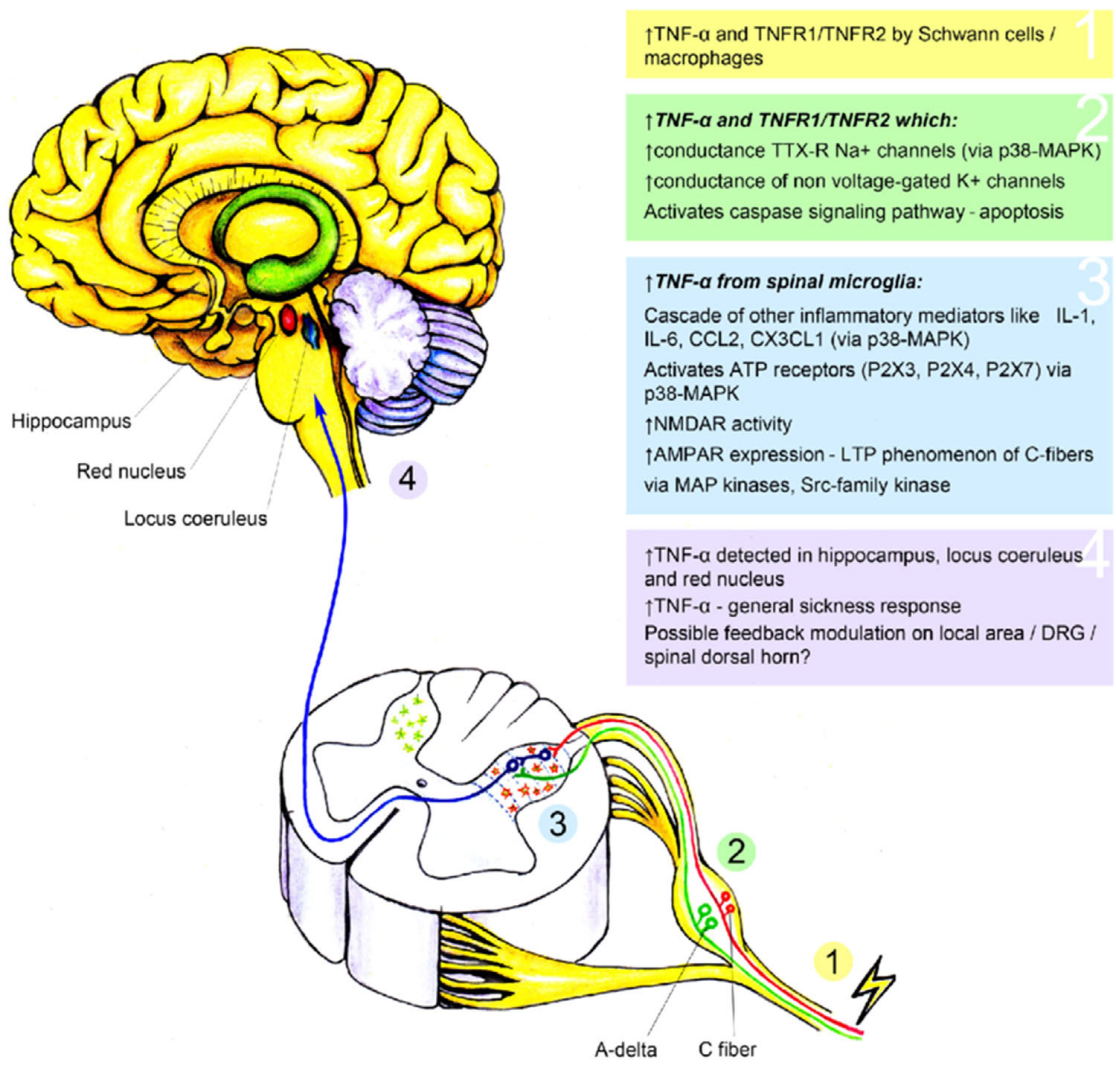
Effects of TNF‐α in the brain and spinal cord (Leung and Cahill 2010).

TNF‐α, a cytokine, plays critical roles throughout the CNS, including in synaptic function, neuroinflammation, and regulation of the immune response. It modulates the expression of neurotransmitter receptors and therefore their ability to modulate synaptic plasticity. Furthermore, TNF‐α regulates oligodendrocyte proliferation, myelin synthesis, and repair, which are all essential for CNS function. High TNF‐α levels are linked to disease progression and symptom severity in neuroinflammatory conditions such as Parkinson's disease (PD), multiple sclerosis (MS), neuro‐Behçet's disease, and Alzheimer's disease (AD). The increase in TNF‐α levels is also related to disease progression and symptom severity. They are also linked to increased depression and chronic pain from TNF. Inhibiting TNF directly has yielded contradictory results in clinical trials, and selective inhibition of certain TNF pathways, which promotes tissue damage by activating immune cells and the production of proinflammatory cytokines, has potential as a potential therapy for TNF‐α (Gonzalez Caldito 2023; Leung and Cahill 2010; McCoy and Tansey 2008; Postal et al. 2016). Figure 6 shows the effects of TNF‐α in the brain and spinal cord in more detail.

TMS alters cognitive function related to the cerebral expression of TNF‐α. TMS affects neuronal excitability which affects inflammation marker such as TNF‐α associated with neuroplastic and cognitive ability. Research shows that low‐frequency rTMS can inhibit inflammatory gene expression of astrocytes and may reduce the TNF‐α level and improve learning ability (Clarke et al. 2021). A systematic review indicated that TMS has beneficial effects on several conditions, including MS, obsessive‒compulsive disorder (OCD), Tourette syndrome (TS), MDD, and autism spectrum disorder (ASD) (Vaishnavi 2023). Additionally, in another study, dementias presented neurophysiological changes linked to diffuse neural damage rather than specific cortical alterations. Under these conditions, TMS has emerged as a promising tool for the assessment of cortical dysfunction, disease progression, and treatment response (Lanza et al. 2022). Nevertheless, the efficacy of rTMS in treating substance use disorders was examined the systematic review level. rTMS has shown significant efficacy in reducing both substance use and craving in those with these disorders (Mehta et al. 2024). In our study, of the 11 studies that measured TNF‐α, 8 reported a significant negative correlation (Boylu et al. 2023; Cha et al. 2022; Liu et al. 2022; Wang et al. 2022; Wu and Liu 2023; Yilmaz et al. 2022; Zhang, Xue et al. 2020; Zhao et al. 2019), whereas 3 reported no significant correlation (Medeiros et al. 2016; Tateishi et al. 2020; Valiuliene et al. 2021). Our data revealed a significant negative correlation between TNF‐α and rTMS intervention (*p* value = 0.000, CI = −10.473 to −2.831). Additionally, we observed a negative correlation between the duration of the intervention and the quantity of TNF‐α (see the chart in Figure 3). The correlation of IL‐6 levels and neuropsychological disorders fits well into neuroinflammation and neurodevelopmental threats convincingly. These results show that high IL‐6 levels themselves can in fact be significantly associated with the increased neuroinflammation which contributes to conditions such as schizophrenia or ASDs. IL‐6 stimulates microglia and astrocytes, which as a result lead to the production of proinflammatory cytokines and neurotoxic substances. Further, the IL‐6 may also be dysregulated, and this will interfere with the blood–brain barrier thus allowing immune cells in the peripheral circulation to access the brain (Kerkis, da Silva Á, and Araldi 2024). Even prenatal exposure to higher IL‐6 concentration has been related to psychiatric disorders in animal models (Couch et al. 2023; Velloso et al. 2022). Moreover, enhanced levels of IL‐6 during early neonatal periods are likely to result in social defects and increased stereotypes. However, there is more information concerning cytokines implicated in neurodevelopmental disorders, and there is a possibility of cytokines’ interaction in causing the disorders (Velloso et al. 2022). Moreover, regarding chronobiological parameters, previous work has shown that IL‐6 levels change during repetitive rTMS, associated with the activity of microglia, the brain's immune cells that release cytokines that modulate synaptic plasticity (Eichler et al. 2023). rTMS‐induced microglial activity can facilitate IL‐6 release and cytokines implicated in plasticity. Innovative studies have indicated that IL‐6 could be a new molecular marker that might be used to determine the effectiveness of rTMS treatment in depression patients (Wang et al. 2022). These variations clearly demonstrate the possibility for using IL‐6 as a biomarker for analysis of the therapy outcomes in depression (Zhou et al. 2023). Our study investigated the impact of rTMS on IL‐6. Four out of eight studies that measured IL‐6 showed a significant negative correlation (Cha et al. 2022; Liu et al. 2022; Wu and Liu 2023; Zhang, Xue et al. 2020), whereas four studies reported no significant correlation (Medeiros et al. 2016; Tateishi et al. 2020; Wang et al. 2022; Yilmaz et al. 2022). We performed a subgroup analysis based on gender differentiation in the IL‐6 meta‐analysis. Nevertheless, no such correlation within each gender group was found. IL‐1β is considered an essential mediator of human neuropsychological disorders due to the connections between neuroinflammation and stress. Moreover, the involvement of IL‐1β has been reported to prevent the alterations of brain damage in several chronic hypoperfusion diseases (Quintana et al. 2021). Currently, IL‐1 receptor antagonists have also been implicated as having the capability of reducing stress‐induced neurogenic deficits thereby giving them the potential in the treatment of depression (Koo and Duman 2009). TMS has variant effects for neuronal excitation that may help modulate inflammatory markers like IL‐1β. In addition to suppressing inflammation, TMS may enhance synaptic plasticity; this is often disrupted by inflammation. TMS has been considered for the management of neuropsychiatric conditions characterized by inflammation such as autism and OCD and for augmenting recovery of neurologic conditions because of the enhancement of cortical plasticity by TMS (Machado et al. 2013; Vaishnavi 2023). Further, TMS has also been described to influence IL‐1β potentially via neuroinflammatory circuits associated with depression and PD. The evidence shows that rTMS reduces glial activation and promotes a pro‐regenerative state apart from the neuroinflammation that takes place in Parkinson's models (60). IL‐1β also modulates neuronal excitability in area of SFO, and TMS may impact these in neurons sensitive to IL‐β (Desson and Ferguson 2003; Han et al. 2023). The detected interleukin receptors in brain allow the TMS regulating directly the IL‐β signaling, thus making utility of TMS as noninvasive treatment in those neurological disorders related to dysregulated IL‐β activity (Haour et al. 1992; Tateishi, Mizoguchi, and Monji 2022). Moreover, in our study, six studies measured the levels of IL‐1β, and all of them were significantly negatively correlated (Boylu et al. 2023; Cha et al. 2022; Liu et al. 2022; Tateishi et al. 2020; Wu and Liu 2023; Zhao et al. 2019). Hs‐CRP is a key biomarker to understanding neuroinflammation and its consequences for neurodegenerative diseases. Systemic inflammation, evidenced by elevated hsCRP levels, is associated with the pathogenesis of AD and vascular dementia (VaD) (Gabin et al. 2018). Additionally, dementia patients have significantly greater hs‐CRP levels than controls, which indicate that hs‐CRP plays a role in cognitive decline (Wang et al. 2012). It may be that hs‐CRP mediates the inflammatory processes that lead to neuronal damage and could therefore serve as a therapeutic target in neurodegenerative disorders (Suleiman Khoury et al. 2024; Tachibana et al. 2024). Although hs‐CRP works in a type of dual manner of neuroinflammation making its role difficult, it does not only work in a destructive mode and hence can offer some protective effects against neuronal injury (Suleiman Khoury et al. 2024). There is a strong correlation between TMS efficacy in the treatment of depression and high sensitivity hs‐CRP levels, controversial as hs‐CRP levels are related to poor outcomes. Some inflammatory markers like hs‐CRP are also increased in patients with MDD and may lower the effectiveness of rTMS (Felger 2019; Rajkumar 2024). Results suggest that baseline hs‐CRP levels are inversely associated with response rates to rTMS (Rajkumar 2024). Nevertheless, rTMS can modulate inflammatory cytokines and seems to alleviate depressive symptoms by that mechanism (Langguth et al. 2005). Patients with lower hs‐CRP levels usually respond better, but some studies show that there is variability, and some patients continue to respond well even with high inflammation (Felger 2019). In our study, four studies measured hs‐CRP: half of them demonstrated a negative correlation (Wang et al. 2022; Zhang, Xue et al. 2020), whereas the other half showed no significant correlation (Cha et al. 2022; Yilmaz et al. 2022).

IFN‐γ implicated in the modulation of synaptic inhibition, neuronal morphology, and the pathogenesis of various neurological conditions. Notably, IFN‐γ enhances inhibitory neurotransmission in neocortical neurons, promoting GABAA receptor activity through protein kinase C pathways (Janach et al. 2022; Janach et al. 2020). Furthermore, maternal immune activation linked to IFN‐γ exposure has been associated with neurodevelopmental disorders, indicating its potential impact on brain development (Pavlinek et al. 2022). On one hand, IFN‐γ can enhance neuroinflammatory responses, leading to detrimental effects such as impaired neurogenesis and increased depressive‐like behaviors (Zhang, He et al. 2020). Conversely, it also exhibits protective functions by regulating astrocytic signaling and suppressing overactive immune responses (Tinkey et al. 2024). IFN‐γ primes microglia, resulting in morphological changes and increased proinflammatory cytokine release (e.g., IL‐1β and TNF‐α). Chronic activation of microglia by IFN‐γ impairs adult hippocampal neurogenesis, contributing to cognitive deficits (Zhang, He et al. 2020). A study indicates that rTMS treatment correlates with clinical responses in depression, highlighting its potential as a biomarker for treatment efficacy (Pathak, Salami, Baillet, Li, and Butson 2013). Furthermore, in our study, two studies measured IFN‐γ: one indicated a negative correlation (Wang et al. 2022), whereas the other showed no significant correlation (Yilmaz et al. 2022).

### 4.3 BDNF

BDNF, neuronal survival and growth factor. Reduced BDNF levels are linked to various brain disorders. Neuroinflammation and proinflammatory cytokines and signaling downregulate BDNF, implicated in Alzheimer's, Parkinson's, Huntington disease, and MS. In Alzheimer's, they noted low BDNF and high proBDNF in Alzheimer's reduces synaptic density implying more neuron death. In PD, reduced BDNF is responsible for motor and motor symptoms resulting from dopaminergic neurons. In Huntington's, low levels of BDNF result in damaging effects to the neurons, and in MS, the BDNF protein offers neuroprotection and repair (Li et al. 2022; Lima Giacobbo et al. 2019). Decreased BDNF levels correlate with increased depressive symptoms and cognitive decline (Correia, Cardoso, and Vale 2023). However, antidepressant therapies can restore BDNF levels, suggesting a therapeutic target (Correia, Cardoso, and Vale 2023). BDNF signaling is linked to stress‐related disorders, with alterations in the BDNF/TrkB system contributing to anxiety (Numakawa and Kajihara 2023). Dysregulation of BDNF affects GABAergic inhibition, which is crucial for anxiety management (Tomoda et al. 2022). Some studies suggest that BDNF gene polymorphisms, for example, Val66Met, can determine response to rTMS and cortical plasticity (Adamson et al. 2022; Parchure et al. 2022). rTMS has been found effective in modulating cognitive functions of schizophrenia and MDD; BDNF is proposed as a marker of rTMS outcome (Su et al. 2023). Genetic variations influencing the action of BDNF are known to result in major differences in the therapeutic outcomes (Parchure et al. 2022). We measured it in our study, six studies. Satisfactorily, rTMS effectively affects inflammatory cytokines and protein factors, suggesting that rTMS is an effective treatment for neurological and neuropsychological diseases. Among them, four showed a positive significant correlation (Bai et al. 2022; Boylu et al. 2023; Valiuliene et al. 2021; Zhao et al. 2019), whereas two studies reported no significant changes (Medeiros et al. 2016; Yilmaz et al. 2022). Additionally, the data synthesis and meta‐analysis revealed a positive correlation between rTMS intervention and BDNF quantity (*p* value = 0.003, CI = 2.8–13.5). Our research matches what others previously reported (Bai, Yang, Chen, and Wang 2023; Eichler et al. 2023). However, their results revealed a significant correlation for IL‐6 compared with our findings. We speculate that the small sample size and high heterogeneity in our meta‐analysis data may be the cause of this discrepancy.

### 4.4 Limitations

Several limitations exist in our study. First, it would have been possible for us to miss relevant studies in languages other than English or databases, as our search was limited to five English electronic databases. Our search strategy focuses exclusively on TNF‐α and rTMS, without exploring other inflammatory cytokines and BDNF. This limits geographical diversity, as the majority of the included studies were conducted in East Asia. Although these studies had small sample sizes and allowed for medication use, this can introduce bias. Unfortunately, most studies used high‐frequency rTMS (10–20 Hz), which precluded our ability to assess the effects of other protocols. As in one study, despite several attempts to contact the authors, TNF‐α levels were not reported (Bai et al. 2022). AEs were not reported by some studies, which hindered complete safety assessment. Both in our meta‐analyses and in other evaluations of intervention effects, there was high heterogeneity, especially for studies with longer intervention durations. Only a few studies exist for IL‐6 and BDNF; thus, we were unable to conduct subgroup analyses. The TNF‐α subgroup with <or = 1 month of intervention time was highly heterogeneous, which is consistent with the variability in demographic and intervention partner characteristics. We failed to perform a meta‐analysis of other inflammatory markers, such as IFN‐γ, hs‐CRP, and IL‐1β, because of a lack of data. Future work should address these limitations via larger, more diverse samples, consistent reporting, and investigations of different rTMS protocols. Finally, a notable limitation of this study is that gender is unevenly distributed over intervention durations. Intervention periods shorter than 1 month mostly studied female participants, whereas longer than 1 month included greater percentage of male participants. Although we found no significant differences in IL‐6 levels in these groups, confirming that gender may not play a major role in determining rTMS intervention pathways, the apparent systematic imbalance in gender distribution across intervention durations creates the potential for a confounding variable. Overlapping gender proportion and intervention duration means that it is hard to tease apart the independent effects of either factor. Our results show no significant differences between the groups but future studies can strive for a more balanced gender representation among groups engaging in the different intervention durations to ensure that there is a more reliable differentiation of effect. This would confirm whether the observed patterns are really independent of gender, or whether there are some biases between gender, treatment duration, and treatment results, which our current analysis cannot detect.

## 5 Conclusion

This systematic review and meta‐analysis examined the effects of rTMS on inflammatory cytokines and BDNF in neuropsychological patients. The findings revealed a significant negative correlation between rTMS intervention and TNF‐α levels, suggesting that rTMS may help reduce inflammation. No significant correlation was found for IL‐6 levels, whereas mixed results were observed for other cytokines like IL‐1β, hs‐CRP, and IFN‐γ. BDNF showed a positive correlation with rTMS, indicating potential neuroprotective effects. The study highlights rTMS as a promising treatment for neurological and neuropsychological diseases by modulating inflammatory markers and neurotrophic factors. This noninvasive technique may offer therapeutic benefits for conditions such as depression, PD, and MS, among others.

## Author Contributions

**Arya Asadizeidabadi**: conceptualization, methodology, resources, writing—original draft, formal analysis, data curation, writing—review and editing, project administration, visualization. **Seyedmohammadamin Hosseini**: methodology, formal analysis, data curation, writing—original draft, writing—review and editing. **Artem Pyatkov**: supervision.

## Disclosure

During the preparation of this work, the author(s) used Claude 3.5 Sonnet to improve readability and language. After using this tool, the authors reviewed and edited the content as needed and take full responsibility for the content of the publication.

## Ethics Statement

This article only includes data from previously conducted and published studies. It does not contain any studies from human participants or animals performed by any of the authors.

## Conflicts of Interest

The authors declare no conflicts of interest.

### Peer Review

The peer review history for this article is available at https://publons.com/publon/10.1002/brb3.70329

## Supporting information



Supporting Information

## Data Availability

The data that support the findings of this study are available from the corresponding author upon request.
