# Supplementary material for: Effects of Repetitive Transcranial Magnetic Stimulation on Tumor Necrosis Factor Alpha in Neuropsychological Disorders: A Systematic Review and Meta‐Analysis
Source: Brain Behav. 2025 Feb 11;15(2):e70329. doi: 10.1002/brb3.70329 (PMC11814483; doi:10.1002/brb3.70329)
Supplement: Supplementary file 1 — Supporting Information [file BRB3-15-e70329-s001.docx]

**Supplementary Materials**

1. **Appendix A**

**Mesh terms**

| **Intervention (TITLE AND ABSTRACT)** | **Outcome (ALL FILDS)** |
| --- | --- |
| ((((((((Magnetic Stimulation, Transcranial) OR Magnetic Stimulations, Transcranial) OR Stimulation, Transcranial Magnetic) OR Stimulations, Transcranial Magnetic) OR Transcranial Magnetic Stimulations) OR Transcranial Magnetic Stimulation, Single Pulse) OR Transcranial Magnetic Stimulation, Paired Pulse) OR Transcranial Magnetic Stimulation, Repetitive) | (((((((((Tumor Necrosis Factor alpha) OR Cachectin-Tumor Necrosis Factor) OR Cachectin Tumor Necrosis Factor) OR TNF-alpha) OR TNFalpha) OR TNF Superfamily, Member 2) OR Tumor Necrosis Factor Ligand Superfamily Member 2) OR Cachectin) OR Tumor Necrosis Factor) |

**Table S1 Search results**

| **Date** | **2/17/2024** |
| --- | --- |
| **Database** | **169** |
| **PubMed/Medline:** | **40** |
| **Scopus:** | **55** |
| **ISI Web of Science:** | **55** |
| **Cochrane** | **18** |
| **https://clinicaltrials.gov/** | **1** |

1. **Appendix B**

**Table S2A RCT JBI tools**

| **Questions/ Study** | **Medeiros et al.** | **Zhao et al** | **Zhang et al.,** | **Liu et al.** | **Wang et al.,** | **Bai et al.,** |
| --- | --- | --- | --- | --- | --- | --- |
| **Was true randomization used for assignment of participants to treatment groups?** | **YES** | **YES** | **YES** | **YES** | **YES** | **YES** |
| **Was allocation to treatment groups concealed?** | **YES** | **N/A** | **N/A** | **N/A** | **YES** | **N/A** |
| **Were treatment groups similar at the baseline?** | **YES** | **YES** | **YES** | **YES** | **YES** | **YES** |
| **Were participants blind to treatment assignment?** | **YES** | **N/A** | **N/A** | **N/A** | **YES** | **N/A** |
| **Were those delivering treatment blind to treatment assignment?** | **YES** | **N/A** | **N/A** | **N/A** | **YES** | **N/A** |
| **Were outcomes assessors blind to treatment assignment?** | **NO** | **N/A** | **N/A** | **N/A** | **NO** | **N/A** |
| **Were treatment groups treated identically other than the intervention of interest?** | **NO** | **YES** | **YES** | **YES** | **YES** | **YES** |
| **Was follow up complete and if not, were differences between groups in terms of their follow up adequately described and analysed?** | **NO / 1 Patient in group rTMS + sham DIMST and 1 patient in sham-rTMS + DIMST because of discontinued intervention** | **YES** | **YES** | **YES** | **YES** | **NO/ 2 patient in treatment group don’t measured blood serum** |
| **Were participants analysed in the groups to which they were randomized?** | **YES** | **YES** | **YES** | **YES** | **YES** | **YES** |
| **Were outcomes measured in the same way for treatment groups?** | **YES** | **YES** | **YES** | **YES** | **YES** | **YES** |
| **Were outcomes measured in a reliable way?** | **YES** | **YES** | **YES** | **YES** | **YES** | **YES** |
| **Was appropriate statistical analysis used?** | **YES** | **YES** | **YES** | **YES** | **YES** | **YES** |
| **Was the trial design appropriate, and any deviations from the standard RCT design (individual randomization, parallel groups) accounted for in the conduct and analysis of the trial?** | **YES** | **YES** | **YES** | **YES** | **YES** | **YES** |

Abbrevation N/A; Not Recoomend

**Table S2B Cohort JBI tools**

| **Questions/ Study** | **Tateishi et al.** | **Valiuliene et al** | **Cha et al** | **Yilmaz et al** | **Wu & Liu** | **Boylu et al.** |
| --- | --- | --- | --- | --- | --- | --- |
| **Were the two groups similar and recruited from the same population?** | **NO** | **YES** | **NO** | **NO** | **YES** | **NO** |
| **Were the exposures measured similarly to assign people to both exposed and unexposed groups?** | **YES** | **YES** | **YES** | **YES** | **YES** | **YES** |
| **Was the exposure measured in a valid and reliable way?** | **YES** | **YES** | **YES** | **YES** | **YES** | **YES** |
| **Were confounding factors identified?** | **YES** | **YES** | **YES** | **YES** | **YES** | **YES** |
| **Were strategies to deal with confounding factors stated?** | **YES** | **YES** | **YES** | **YES** | **YES** | **YES** |
| **Were the groups/participants free of the outcome at the start of the study (or at the moment of exposure)?** | **YES** | **YES** | **YES** | **YES** | **YES** | **YES** |
| **Were the outcomes measured in a valid and reliable way?** | **YES** | **YES** | **YES** | **YES** | **YES** | **YES** |
| **Was the follow up time reported and sufficient to be long enough for outcomes to occur?** | **YES** | **YES** | **YES** | **YES** | **YES** | **YES** |
| **Was follow up complete, and if not, were the reasons to loss to follow up described and explored?** | **YES** | **NO/ control group didn’t follow up as exposure group** | **YES** | **NO/ 1 month after exposure 8 patient didn’t measured** | **YES** | **YES** |
| **Were strategies to address incomplete follow up utilized?** | **YES** | **NO** | **YES** | **YES** | **YES** | **YES** |
| **Was appropriate statistical analysis used?** | **YES** | **YES** | **YES** | **YES** | **YES** | **YES** |

Abbrevation N/A; Not Recoomend

1. **Appendic C**

**
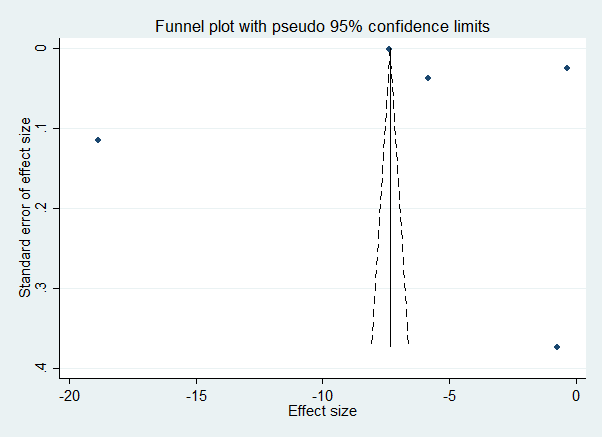
**

**Fig. S1 Funnel plot of TNF meta-analysis**


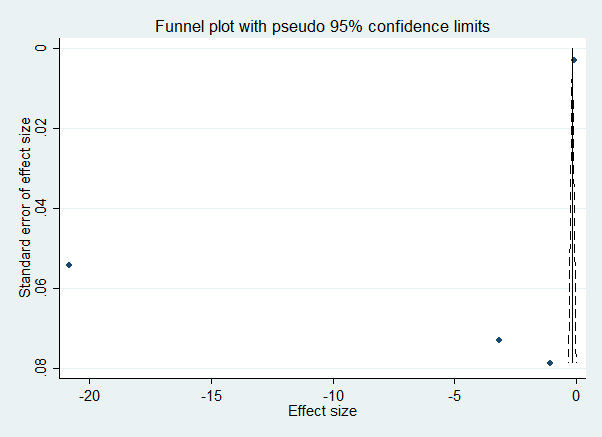


**Fig. S2 Funnel plot of IL-6 meta-analysis**

**
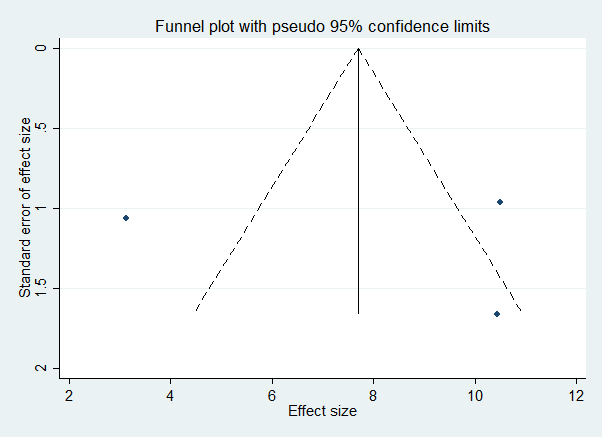
**

**Fig. S3 Funnel plot of BDNF meta-analysis**

1. **Appendix D**

**Table S3 Publication bias Egger test**

| publication bias |  |
| --- | --- |
| Inflammatory cytokines | Egger |
| TNF-α | P = 0.539 |
| IL-6 | P = 0.352 |
| BDNF | P = 0.910 |

**Table S4 Sensitivity test**

|  | Sensitivity |
| --- | --- |
| TNF-α | Liciane F et al \| -0.814 -1.668 0.040 |
